# Phosphate of Quinine

**Published:** 1831

**Authors:** 


17. *Phosphate of Quinine.*—Dr. Harless, of Bonn, has discovered that the phosphate of quinine is not only more agreeable to the palate than the sulphate, but that by reason, the union of the vegetable alkali with an animal acid (the phosphoric) the medicine is better suited to the purpose of animalization; it combines more readily with the chyme and chyle, and hence is more properly conveyed to every part of the system. The phosphate is best suited to irritable stomachs, and is vastly superior to any other preparation of quinine in such cases. Dose from one to four grains.—*Journ. de Chim. Med.*

